# Signal transduction in primary human T lymphocytes in altered gravity – results of the MASER-12 suborbital space flight mission

**DOI:** 10.1186/1478-811X-11-32

**Published:** 2013-05-07

**Authors:** Svantje Tauber, Swantje Hauschild, Claudia Crescio, Christian Secchi, Katrin Paulsen, Antonella Pantaleo, Angela Saba, Isabell Buttron, Cora Sandra Thiel, Augusto Cogoli, Proto Pippia, Oliver Ullrich

**Affiliations:** 1 Institute of Anatomy, Faculty of Medicine, University of Zurich, Winterthurerstrasse 190, Zurich, CH-8057, Switzerland; 2 Department of Biomedical Sciences, University of Sassari, Via Muroni, 25, Sassari 07100, Italy; 3 Department of Machine Design, Engineering Design and Product Development, Institute of Mechanical Engineering, Otto-von-Guericke-University Magdeburg, Universitätsplatz 2, Magdeburg, D- 39106, Germany; 4 Department of Medicine, University of Verona, Piazzale A. Stefani 1, Verona 37126, Italy; 5 Zero-G Life Tec, Riedhofstrasse 273, Zurich 8049, Switzerland; 6 Zurich Center for Integrative Human Physiology (ZIHP), University of Zurich, Zurich, Switzerland

**Keywords:** T cell activation, Microgravity, Gravi-sensitivity, Hypergravity, Space flight

## Abstract

We investigated the influence of altered gravity on key proteins of T cell activation during the MASER-12 ballistic suborbital rocket mission of the European Space Agency (ESA) and the Swedish Space Cooperation (SSC) at ESRANGE Space Center (Kiruna, Sweden). We quantified components of the T cell receptor, the membrane proximal signaling, MAPK-signaling, IL-2R, histone modifications and the cytoskeleton in non-activated and in ConA/CD28-activated primary human T lymphocytes. The hypergravity phase during the launch resulted in a downregulation of the IL-2 and CD3 receptor and reduction of tyrosine phosphorylation, p44/42-MAPK phosphorylation and histone H3 acetylation, whereas LAT phosphorylation was increased. Compared to the baseline situation at the point of entry into the microgravity phase, CD3 and IL-2 receptor expression at the surface of non-activated T cells were reduced after 6 min microgravity. Importantly, p44/42-MAPK-phosphorylation was also reduced after 6 min microgravity compared to the 1g ground controls, but also in direct comparison between the in-flight μg and the 1g group. In activated T cells, the reduced CD3 and IL-2 receptor expression at the baseline situation recovered significantly during in-flight 1g conditions, but not during microgravity conditions. Beta-tubulin increased significantly after onset of microgravity until the end of the microgravity phase, but not in the in-flight 1g condition. This study suggests that key proteins of T cell signal modules are not severely disturbed in microgravity. Instead, it can be supposed that the strong T cell inhibiting signal occurs downstream from membrane proximal signaling, such as at the transcriptional level as described recently. However, the MASER-12 experiment could identify signal molecules, which are sensitive to altered gravity, and indicates that gravity is obviously not only a requirement for transcriptional processes as described before, but also for specific phosphorylation / dephosphorylation of signal molecules and surface receptor dynamics.

## Background

Gravity has been a constant force throughout evolutionary history on Earth. Thus, one of the fundamental biological questions is how the architecture and function of human cells are related to the gravitational force and thereby adapted to life on Earth. The cells of the human immune system are especially sensitive to altered gravity, and it is for this reason that they comprise a superior biological model system to investigate if the earth’s gravity is important for signal transduction processes inside mammalian cells.

Beginning already in the early days of human spaceflight, an enhanced susceptibility to infections has been observed among astronauts and frequently reported on: During space flights or very soon after returning to the earth astronauts of the Apollo missions suffered from bacterial and viral infections [1,2], while latent viruses such as varicella zoster were also reactivated [3,4]. Initial evidence of disturbed cellular function appeared during investigations of lymphocytes from astronauts of the Soyuz and Skylab missions, who exhibited a markedly decreased response to mitogenic stimulation during and after space as compared to the status before flight [5,6]. Subsequent *in vitro* experiments during the first Spacelab-Mission could demonstrate that the proliferative response of lymphocytes to mitogenic stimulation was strongly impaired under space conditions [7].

This impairment of lymphocyte activation and the resulting immune deficiency is discussed as a serious limitation for manned long-term space flights [8], where not only the extraordinary psychological stress in a confined environment and the enhanced radiation, but also the reduced gravity represent a major and direct “stress factor” at the cellular level. Because many studies have revealed strong effects of microgravity on immune cell function [9-11], microgravity is now considered as one of the major causes of immune dysfunction during space flight. Experiments were performed during manned space flights, on board of orbital, suborbital (sounding rockets) and parabolic flights, and supported by ground-based model systems for simulated microgravity such as clinorotation, 3D-random positioning machines and diamagnetic levitation [12]. Experiments using real as well as simulated microgravity revealed several microgravity-induced alterations in non-activated and activated T lymphocytes. Gravity-sensitive functions of T lymphocytes comprised cell cycle regulation [13], epigenetic regulation [14], chromatin regulation [15], expression profile of microRNA [16], cell motility [17] and regulation of apoptosis [18]. Furthermore, expression of cytokines such as interleukin-1, interleukin-2, interferon-gamma and tumor necrosis factor changed in microgravity [19]. Experiments using ConA-activation of T lymphocytes during a space shuttle flight revealed that microgravity altered protein kinase C distribution [20]. In random positioning machine (RPM) experiments, expression of early genes of T lymphocyte activation, which are regulated primarily by transcription factors NF-κB, CREB, ELK, AP-1, and STAT, were downregulated [21]. Interestingly, mRNA of IL-2 and IL-2 receptors were down-regulated in ConA-activated T lymphocytes during clinorotation [22] and in the random positioning machine [12] within the range of hours. Because the IL-2 receptor transduces major proliferative signals in activated T cells, the diminished proliferative response of T cells upon stimulation during microgravity [7,9] could also be caused by a reduced expression of IL-2, resulting in an impairment of positive regulatory feedback loops. Thus, whereas the phenomenon of reduced activation of T cells during microgravity is well described and verified [5,7,9,12-22], the exact molecular mechanisms are not elucidated.

Recent studies focused on the effect of altered gravity at the level of gene transcription [13,21,22]. In addition, possible mechanisms for rapid gravisensitive effects on the regulation of gene expression were discussed [13]. Because transcriptional effects of microgravity could appear within less than one minute [13], there is the possibility that the molecular architecture of signal modules, including cell surface receptors, is altered within a few minutes. It is also not yet understood if and how microgravity interferes with the events of T lymphocyte activation, the membrane proximal and cytoplasmatic signal transduction cascades and the IL-2/IL-2R activation loop. Because microgravity affected the protein kinase C [20], whereas delivery of first activation signal, patching and capping of conA-binding membrane proteins occurred normally [9], the existence of gravi-sensitive cellular targets could be assumed upstream from PKC and downstream from the TCR/CD3, where the lipid-raft-associated signalosome complex is located.

We therefore investigated the influence of altered gravity on key proteins of T cell activation. We quantified components of the T cell receptor, the membrane proximal signaling, MAPK-signaling, IL-2R, histone modifications and the cytoskeleton in non-activated and in ConA/CD28-activated primary human T lymphocytes. The experiments were performed during the MASER-12 ballistic suborbital rocket mission of the European Space Agency (ESA) and the Swedish Space Cooperation (SSC) at ESRANGE Space Center (Kiruna, Sweden). In early times, many cell biology experiments in space have suffered from a lack of adequate controls (e.g., 1g control centrifuges) and proper experimental conditions (such as well-controlled temperature) [19]. Our experiment hardware consisted of an on-board reference centrifuge (for the 1g group) and automated activation and fixation devices for the cell samples. In addition to the μg and 1g samples (which were activated at the onset of μg and fixed after 6 min), baseline samples were fixed directly before entry into the μg phase of the ballistic flight. An identical hardware module was used for ground control experiments. Due to the technical (limitation of cell numbers and culture volumes) and logistical (experiment preparation, execution and sample recovery at the Arctic circle) requirements, FACS analysis was chosen as the most appropriate method for quantification of a wide range of signal molecules in a small sample size. The number of experimental groups, the group size and the number of different analysis parameters were given by the technical conditions and were strictly limited by the maximal cell number. Analysis was performed with a broad focus on key signal pathways and molecular alterations during T cell activation.

Cell biology experiments in space should be also accompanied or preceded by a program of ground-based investigations and experiments in parabolic flights whenever possible [19]. Thus, extensive ground based experiments have been performed under the ESTEC Contract nr 20562/07/NL/VJ (ESA-CORA-GBF-2005-005): In experiments with a fast rotating 2D clinostat, we detected strong and rapid initial MAPK activation [15] and an enhanced expression of p21 Waf1/Cip1 protein [13] in human T cells within minutes of clinorotation. During parabolic flight experiments, mRNA expression of the cell cycle arrest protein p21 increased 4.1-fold after 20s real microgravity in primary CD4+ T cells and 2.9-fold in Jurkat T cells, compared to 1 g in-flight controls after CD3/CD28 stimulation [13]. Ground-based studies to investigate the effect of hypergravity (1.8g and 9g) did not reveal any effect on cell cycle control signaling, MAPK signaling, transcription factor activity or epigenetic alterations up to 15 min.

The MASER-12 experiment investigated the influence of altered gravity on key proteins of T cell activation and signaling and identified signaling events which are sensitive to altered gravity.

## Materials and methods

### MASER-12 mission profile

**Figure 1:**
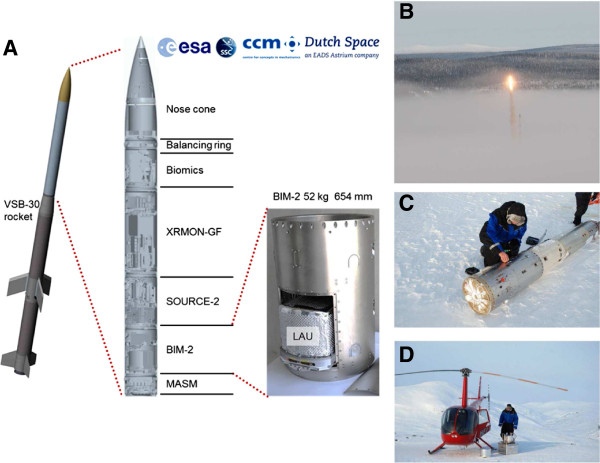
**Payload configuation of MASER-12, launch and recovery. A.** Payload configuration of the MASER-12 sounding rocket. MASER-12 consists of a VSB-30 motor (S-30 solid rocket stage engine with a S-31 second stage engine) and of the payload. MASER-12 hosted five experiments and provided six minutes microgravity. The experiment of the study was performed in the module “Biology In Microgravity“(BIM-2) where the LAU (“Late Access Unit“) containing the isolated cells was installed shortly before the launch. (Pictures were kindly provided by SSC). **B.** MASER-12 was launched on February 13th 2012, 09:32 am, from the ESRANGE Space Centre near Kiruna, Sweden, north of the Arctic circle). **C.** Work with BIM-2 (Biology In Microgravity-2) module at the landing spot. The recovery of the LAU was successful. The module was switched off, by access from the umbilical connector, at 1 hour 20 minutes after lift-off. **D.** The Late Access Unit (LAU) was transported back in a dedicated helicopter from the landing spot.

MASER-12 consists of a VSB-30 motor (S-30 solid rocket stage engine with a S-31 second stage engine) and of the payload (Figure 1A) and was launched on February 13^th^ 2012, 09:32 am, from the ESRANGE Space Centre near Kiruna, Sweden, north of the Arctic circle (Figure 1B). During the ballistic suborbital flight, an altitude of 260 km and 6 min of microgravity were achieved. Further profile parameters were: nominal diameter 17 inches, mass 389.1 kg, apogee 259.8 km, microgravity time 390 sec, landing site distance 104.8 km, landing site azimuth 338°.

### Experiment module BIM-2

**Figure 2:**
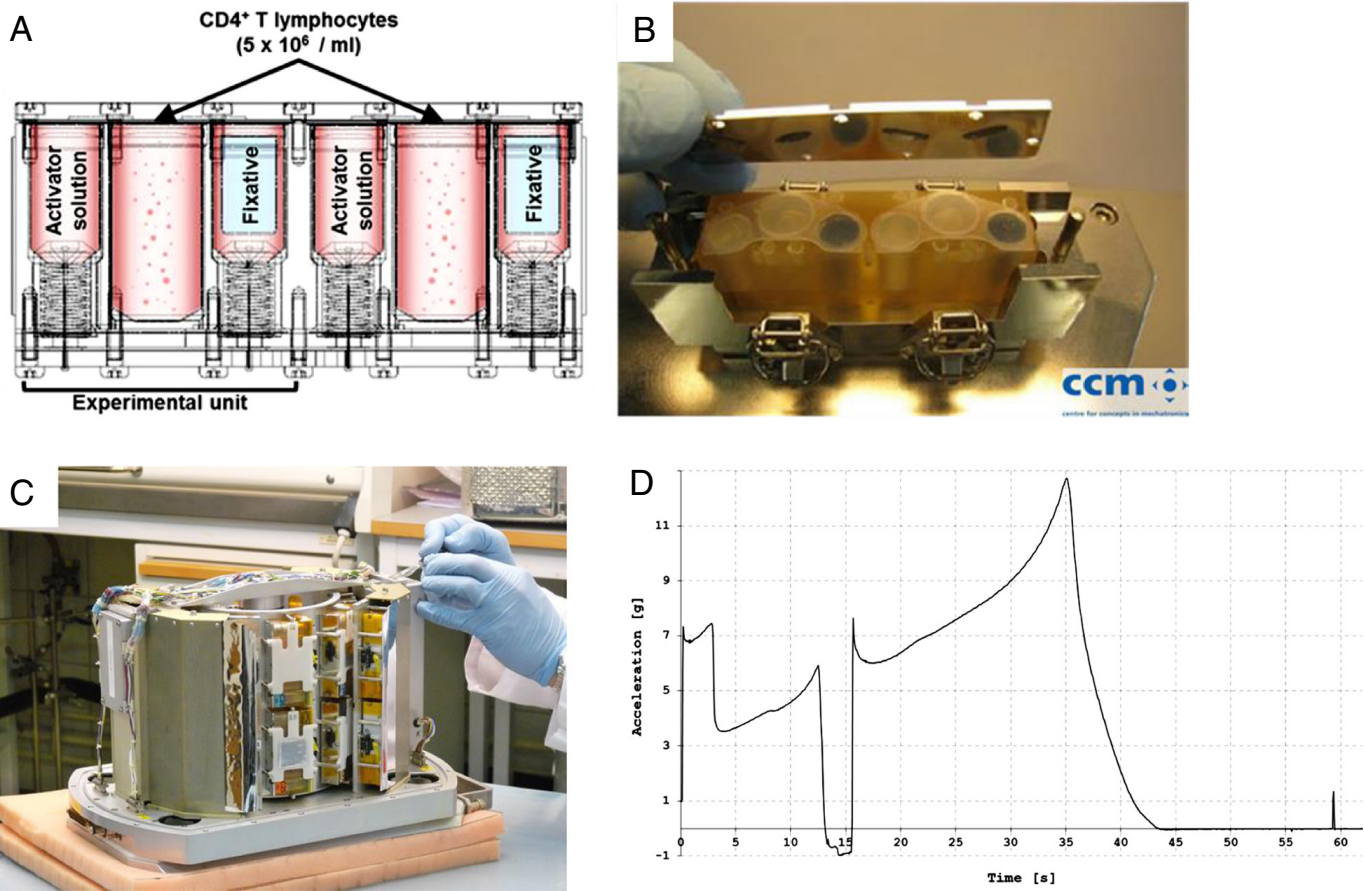
**Experiment module BIM-2. A.** “Mix Unit” flight hardware cassette used for the experiment on the sounding rocket MASER-12. As depicted in the schematic illustration one hardware cassette hosts two experimental units. One experimental unit contains three chambers made of a flexible membrane: one main chamber for cell suspension and two storage chambers, one being the activator solution and one for the fixative. Releasing the tension from the springs beneath the activator and the fixative chamber pushes the solutions into the main chamber (also called reaction chamber) via little tubes in the lid. The picture was kindly provided by CCM, Nuenen, Netherlands. **B.** Picture of one hardware cassette. The picture was kindly provided by CCM, Nuenen, Netherlands. **C.** Late Access Unit (LAU). The main components of the LAU are a reference centrifuge incorporated in the middle and two static racks (μg rack) along the edge. These two components carry the flight hardware cassettes containing cell suspensions and reaction solutions. **D.** Acceleration profile of MASER-12 during the launch. Almost 13 g were detected during MASER-12 launch. The picture was kindly provided by Swedish Space Corporation (SSC).

The experiment was performed in the flight module “Biology In Microgravity 2” (BIM-2) that has been developed by Swedish Space Corporation (SSC) and Dutch Space and Centre for Concepts of Mechatronics (CCM). This module hosted the “Late Access Unit” (LAU), which was installed into the payload 4 hours before launch (Figure 1A). An integrated thermal bath maintained the experiment temperature at 36.5 ± 0.5°C. The LAU contained the hardware cassettes/devices that were filled with the isolated primary human T cells for the experiment. Those cassettes/devices were either installed on a reference centrifuge that provided 1g or on a static rack (μg Rack) so that the cells experienced real microgravity during the flight (Figure 1 and Figure 2C). Besides the LAU on the rocket, another unit, which also hosted hardware cassettes/devices for the 1g hardware control samples, was kept at 37°C in an incubator on ground.

T lymphocytes, activator solution and fixative were loaded into the flight hardware “Mix units” that have been developed by CCM, Nuenen, Netherlands (Figure 2AB). A hardware cassette hosts two experimental units of which each unit contains three chambers. The two outer chambers were connected via tiny tubes in the lid with the middle chamber. The chambers for the fixative contain special storage compartments, since fixatives for biological material diffuse easily through rubbers and plastics. Storage compartments consisted of fluorocarbon elastomer (Viton®), demonstrated to be resistant against formaldehyde in biocompatibility experiments.

Springs under tension are localized beneath the activator and the fixative chamber. Those springs are automatically released at preset time points, so that the solutions in the chambers are pushed via the connecting tubes into the reaction chamber. The activator solution was transferred at the onset of microgravity, whereas the fixative was transferred either at the onset of microgravity (for baseline control experiments) or after 5 min microgravity before re-entry into the Earth’s atmosphere. Vibration tests with the BIM-2 module, centrifuge tests and tests with dummies were performed during flight qualification procedures. Accelerations of the payload and of the centrifuge were continuously monitored and recorded during flight.

### Isolation of primary human T lymphocytes

The isolation of CD4+ T lymphocytes was performed using two male voluntary blood donors (Kiruna Hospital, Sweden) by density gradient centrifugation using HISTOPAQUE®-1077 (Sigma) solution with a density of 1.077 g/mL according to the supplier’s protocol. The blood has been provided by the blood bank at the Kiruna hospital under Swedish law and ethics regulations. The blood has been drawn by the hospital Kiruna from healthy voluntary blood donors. Voluntary donors provided written, informed consent before blood donation. The study was conducted in accordance with Good Clinical Practice guidelines and the Declaration of Helsinki. The blood was carefully layered on HISTOPAQUE®-1077 (Sigma) in a ratio of 1:1. After centrifugation at 400g for 30 min at room temperature, the upper plasma phase was removed. The layer of peripheral blood mononuclear cells (PBMCs) that was formed at the plasma-HISTOPAQUE®-1077 interface was collected. Afterwards, PBMCs were washed twice at 250 g for 10 min (RT) with Hank’s Balanced Salt Solution (HBSS, Sigma). Supernatant was removed and the erythrocytes were lysed by adding Tris-buffered ammonium chloride (ACT) for 10 min. ACT was inactivated with HBSS. PBMCs were washed and resuspended in serum-free RPMI 1640 medium (Invitrogen). Isolation of CD4+ T lymphocytes from PBMCs was performed by Human T Cell Enrichment Columns (R&D systems). T cells were counted and viability was determined by the Trypan Blue exclusion test (Sigma).

### Experimental setup

**Figure 3:**
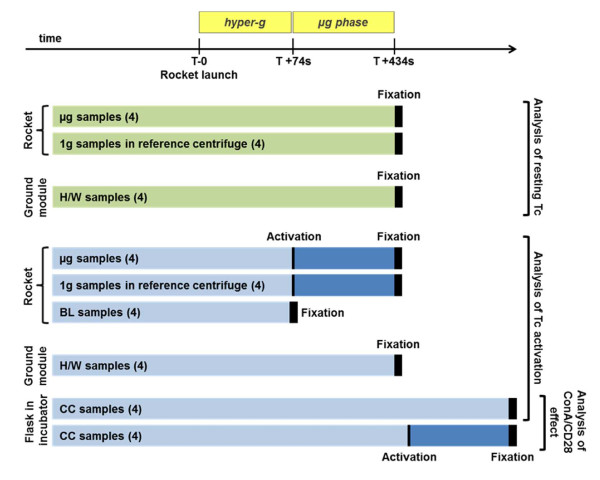
**Experimental design during the MASER-12 mission.** For the analysis of resting T cells three different gravity conditions were tested: μg samples which were installed on the μg Rack in the LAU on board of the rocket, 1g samples that were installed on the on-board reference centrifuge providing 1g, and H/W samples that were installed in the hardware ground module analogue to the flight hardware. With re-entry of the payload into the Earth’s atmosphere all samples were automatically fixed. The analysis of T cells activation involved five different conditions. In addition to the μg and the 1g samples, baseline (BL) samples were used, which were fixed before onset of microgravity (μg phase). At this stage, the μg samples and the 1g samples were activated. The fixation of those two conditions were executed parallel with the appropriate H/W samples, although these were not activated. The fifth condition consisted of culture control (CC) samples, where T cells were cultured under normal culture conditions. Those samples were fixed manually a few minutes after rocket launch due to safety reasons.

During the experiment, the effect of altered gravity on non-activated and activated T cells was investigated (Figure 3). Purified CD4+ T lymphocytes were activated with 10 μg/ml Concanavalin A (ConA, Sigma) and 4 μg/ml.anti CD-28 (BD Bioscences). The experimental setup for non-activated T cells consisted of the experimental group’s hardware control (H/W), the on board 1g control (1g) and the micro-g samples (μg). The experimental setup for T cell activation additionally contained baseline controls (BL) and cell culture controls (CC). Each experimental group consisted of four samples. Analysis of non-activated T cells: CD4+ T lymphocytes isolated from one single blood donor were used. Samples were prepared as follows: four samples were placed onto the μg Rack (μg samples) and four samples were placed into the on-board reference centrifuge (1g samples). Furthermore, four samples were loaded into a hardware module (H/W samples) that was kept on ground as a 1g ground control. All samples were fixed at the same time after 6 min μg and before re-entry into the Earth’s atmosphere. Analysis of activated T cells: CD4+ T lymphocytes isolated from one single blood donor were used. The analysis consisted of five experimental conditions with four samples each. In the first condition the cells were kept at normal culture conditions on ground (CC samples, cell culture flasks, 37°C, 5% CO2) and in the second condition the cells were cultured in the ground hardware module (H/W samples), identical to the LAU on board of MASER-12. For the third condition samples were fixed after launch before onset of the microgravity (BL samples), so that they provided information of the influence of the hypergravity phase (up to almost 13g, Figure 2D). For 1g and μg conditions, samples were installed in the 1g on-board reference centrifuge or in the μg rack of the LAU respectively. Those two samples groups were activated parallel to each other at the onset of the μg phase. The H/W samples as well as the 1g and the μg samples were also automatically fixed parallel to each other after 6 min μg and before re-entry into the Earth’s atmosphere. The fixation of the CC samples were performed manually.

### Implementation of the experiment in the BIM-2 hardware

The main chambers (reaction chambers) were filled with 1 mL of CD4+ T lymphocyte solution (5 × 10E6 cells/mL). The activator solution containing Concanavalin A (ConA, Sigma) and anti-CD28 monoclonal antibody (BD Biosciences) was prepared as a stock solution with concentrations of 44 μg/mL and 18 μg/mL, respectively. In order to reach a final concentration of 10 μg/mL ConA and 4 μg/mL anti-CD28 in the reaction chamber, 700 μL of the stock solution were filled in the intended chambers. Control solution (medium) was used instead of activator solution for samples that were not activated. To reach a final concentration of 1.23% formaldehyde for fixation of the cells, 400 μL of 20% formalin (7.4% formaldehyde) were filled into the special storage compartments in the appropriate chambers. The space between the chamber and the storage compartment was filled with 300 μL medium. During the flight, the springs in the hardware devices were automatically released at particular time-points to either transfer the activator solution or the fixative into the reaction chamber (Figure 2A).

### Sample recovery and processing

The LAU was brought back to ESRANGE Space Centre approximately two hours after the launch (Figure 1CD). The hardware cassettes/devices were directly removed from the LAU and inspected by CCM and Dutch Space. Afterwards, samples were transferred to 15 mL tubes (Bioswisstec) and centrifuged at 300 g for 5 min. Cells were resuspended in 2 mL Stain Buffer (BD Biosciences), transferred to 2 mL test tubes and either analyzed on-site or stored at 4°C until transport from ESRANGE Space Center to the University of Zurich. On-site-analysis during mission conditions at ESRANGE Space Port was performed using the CyFlow® SL (Partec, Muenster). Three samples had to be excluded from further analyses because automatic transfer of the activator or the fixative solution into the cell chamber was not complete.

### Flow cytometry analysis

After retrieval from the rocket and the ground control units, samples were centrifuged (300g, 5 min), supernatant was removed and cells were resuspended in Stain-buffer (BD). At this step, cells for FACS-analysis of unstained cells were branched off. For staining of CD3 and IL-2R samples were not permeabilized (surface staining); for all other stainings cells were permeabilized (intracellular staining) with Perm Buffer III (BD) according to the manufacturer’s instructions. Cells were centrifuged, supernatant was removed and cells were resuspended in 80 μl Stain Buffer containing Alexa Fluor® 488-conjugated antibodies. Antibodies were used in the following dilutions: CD3 (PromoCell, PK-AB913-144), 1:80; IL-2R (PromoCell,PK-AB913-104), 1:40; β-Tubulin (Cell Signaling, 3623), 1:640; Phospho-p44/42 MAPK (Erk1/2) (Thr202/Tyr204) (D13.14.4E) (Cell Signaling, 4344), 1:160; Phospho-p44/42 MAPK (Erk1/2) (Thr202/Tyr204) (Cell Signaling, 4374), 1:160; α-Tubulin (Cell Signaling, 8058), 1:640; Zap-70 (D1C10E) (Cell Signaling, 9473), 1:80; Acetyl-Histone H3 (Lys9) (Cell Signaling, 9683), 1:400; Phospho-Histone H3 (Cell Signaling 9708), 1:40; Vimentin (Cell Signaling, 9854), 1:320; Mouse anti-LAT (pY171) (BD Bioscience, 558519), 1:8; Mouse (MOPC-21) mAb IgG1 Isotype Control (Cell Signaling, 4878), 1:80; Rabbit (DA1E) mAb IgG Isotype Control (Cell Signaling, 2975), 1:40. Mouse anti-human interleukin-2 receptor and mouse anti-human CD3 both coupled to PromoFluor-488 Premium were custom PromoKine formulations (PromoCell, Heidelberg, Germany). After 30 min of incubation, 250 μl Stain Buffer were added, and cytometry was carried out using a FACSCanto II (BD) or the CyFlow SL (Partec) with 488nm solid state laser.

For data analysis FlowJo software (TreeStar) was used. Only cells that appeared single and alive according to forward- and sideward-scatter were analyzed. Results are expressed as the relative fluorescent intensity (RFI) which is calculated by dividing the geometric median fluorescent intensity (MFI) of the test antibody by the MFI of the isotype-and species-matched unspecific control antibody.

### Statistical analysis

Comparison of the two groups was carried out using the Mann-Whithney U test for non-parametrical comparison. Analyses were made using GraphPad Prism 5.0 software. p < 0.1 was considered to be statistically significant.

## Results

### Surface expression of CD3

**Figure 4:**
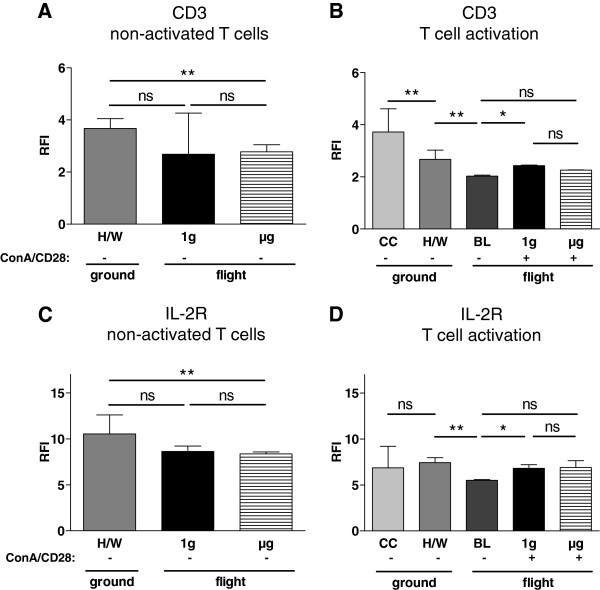
**CD3 and IL-2 receptor surface-expression of T lymphocytes exposed to different gravity conditions measured by FACS-analysis.** Non-activated and Concanavalin A (ConA)/CD28-activated CD4+ T lymphocytes were exposed to altered gravity. **A** and **B**. Samples were stained against surface-CD3 and analyzed by FACS; data are expressed as relative fluorescence intensity (RFI). **A:** Non-activated T lymphocytes exposed to microgravity (μg) showed a significant reduction of CD3 surface expression in comparison to the cells of the flight hardware samples on ground (H/W). **B:** ConA/CD28 activated (+) T lymphocytes at 1g (on-board reference centrifuge) showed a significantly higher expression of CD3 compared with the cells of the baseline (BL) samples, while the μg samples did not show a significant higher level compared to BL samples. In hardware (H/W) controls the CD3 signal was higher than in BL samples, while culture control (CC) samples showed the highest signal of all experimental groups. Medians and interquartile ranges are shown (* p < 0.1, ** p < 0.05, ns = not significantly different, two-tailed Mann–Whitney-U-Test). C and D. Samples were stained against surface-IL-2R and analysed by FACS; data are expressed as relative fluorescence intensity (RFI). **C:** Non-activated (−) T lymphocytes exposed to microgravity (μg) showed a significant reduction of IL-2R in comparison to the cells of the flight hardware on ground (H/W). **D:** ConA/CD28 activated (+) T lymphocytes at 1g conditions (on-board reference centrifuge) (1g) showed a significantly higher expression of IL-2R compared with the cells of the baseline (BL) samples. In hardware (H/W) samples the IL-2R signal was higher than in the BL samples. No significant difference was found between culture controls (CC) and H/W samples. Medians and interquartile ranges are shown (* p < 0.1, ** p < 0.05, ns = not significantly different, two-tailed Mann–Whitney-U-Test).

The first step of T-cell activation is binding, and recognition of the antigen by the TCR/CD3 is complex. Therefore, surface TCR/CD3 was quantified as the first component of the signaling cascade. The analysis of non-activated T cells revealed that the fluorescence intensity of CD3 was not significantly different between the 1g (2.68, 2.52-4.26, n = 3) and the μg samples (2.78, 2.53-3.05, n = 4) (p > 0.9999). CD3 expression in the μg samples and in the 1g samples was lower than in the H/W samples (3.67, 3.38-4.05, n = 4). This difference was tested significantly only in case of the μg samples (p = 0.0286) (μg samples vs. H/W samples p = 0.5714) (Figure 4A). The highest level of surface CD3 was found in CC samples (3.72, 3.55-4.39, n=4) (Figure 4B). The expression in H/W samples was significantly lower (2.67, 2.43-2.95, n=4, p=0.029). In BL samples the level was even further decreased compared to H/W (2.025, 1.945-2.068, n = 4). During T cell activation, no difference could be detected between the 1g samples (2.43, 2.20-2.45, n = 3) and the μg samples (2.26, 1.84-2.27, n = 3, p = 0.4000). The comparison of the 1g and the μg samples with the BL samples revealed a significantly higher CD3 level in the 1g samples (p = 0.0571) but not in the μg samples (p = 0.6286) (Figure 4B).

### Surface expression of IL-2R

Functionality of IL-2R/IL-2 surface expression is a prerequisite for full T cell activation [23,24]. The status of surface IL-2R was quantified to assess the functional condition of IL-2/IL2R signalling in the early phase of T cell activation under microgravity. In the analysis of non-activated T cells, no difference was detected in the IL-2R expression between the 1g samples (8.64, 8.05-9.21, n = 3) and the μg samples (8.37, 6.34-8.57, n = 4, p = 0.4000). In the μg samples the level of IL-2R was significantly lower than in the H/W control samples (10.54, 9.15-12.61, n = 4, p = 0.0286), whereas IL-2R in the 1g samples was also lower than in the H/W samples, although this was not significant (p = 0.1143) (Figure 4C).

In the analysis of T cell activation, no significant difference in IL-2 expression between 1g samples (6.81, 6.72-7.21, n = 3) and μg samples (6.90, 5.11-7.64, n = 3) was detected (p = 0.9000). Compared to the BL samples (5.51, 5.18-5.58, n = 4) 1g and μg samples show enhanced IL-2R expression, but only the difference between 1g samples and BL samples could be tested with statistical significance (p = 0.0571) (BL vs. μg, p = 0.4000). A significant decrease in the IL-2R signal was observed comparing the H/W (7.43, 6.81-7.93, n=4) samples with the BL samples (p=0.0286), whereas the CC samples (6.86, 5.823-8.800) did not differ from the H/W samples (Figure 4D).

### Expression of ZAP 70

**Figure 5:**
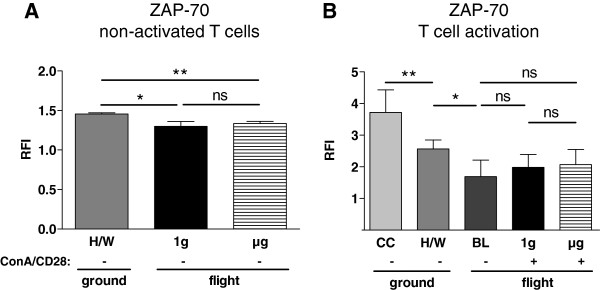
**ZAP 70-expression of T lymphocytes exposed to different gravity conditions measured by FACS-analysis.** Non-activated and Concanavalin A (ConA)/CD28-activated CD4+ T lymphocytes were exposed to altered gravity. Samples were stained against ZAP 70 and analyzed by FACS; data are expressed as relative fluorescence intensity (RFI). **A:** Non-activated (−) T lymphocytes exposed to microgravity (μg) and to an on-board reference centrifuge (1g) showed a significant reduction of ZAP 70 staining compared to the cells of the hardware ground control (H/W). ZAP 70 staining was not significantly different between 1g and μg samples. **B:** ZAP 70 staining of ConA/CD28 activated (+) T lymphocytes was neither significantly different between 1g and μg samples, nor between baseline (BL) samples and 1g or μg samples. The ZAP 70 staining was significantly decreased between culture controls (CC) and H/W samples, and between H/W samples and baseline (BL). Medians and interquartile ranges are shown (* p < 0.1, ** p < 0.05, ns = not significantly different, two-tailed Mann–Whitney-U-Test).

In non-activated T cells a reduction of ZAP 70 staining was observed between H/W ground controls (1.46, 1.39-1.47, n= 4) and 1g samples (1.30, 1.19-1-36, n=3, p=0.057), as well as between H/W controls and μg samples (1.34, 1.32-1.37, n=4, p= 0.029). Direct comparison between μg samples and 1g samples did not reveal a significant difference (p=0.400) (Figure 5A). Comparison of ZAP 70 staining showed a significantly lower level in hardware ground controls (2.57, 2.18-2.80, n=4) compared to culture controls (3.72, 3.62-4.26, n=4, p= 0.0286) (Figure 5B). Baseline samples (1.69, 1.65-2.09, n=4) had a significantly lower staining than hardware ground controls (p=0.057).

During T cell activation, no differences were observed when comparing staining intensities in baseline samples with either μg samples (2.07, 1.89-2.55, n=3, p=0.200) or 1g samples (1.98, 1.83-2.39, n=3, p=0.200). Likewise, direct comparison of μg with 1g samples revealed no significant difference (p=0.700) (Figure 5B).

### Phosphorylation of LAT

**Figure 6:**
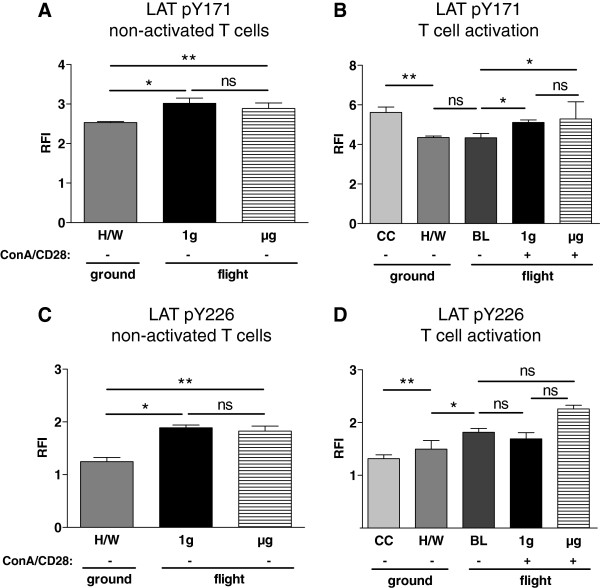
**pY171-LAT and pY266-LAT in T lymphocytes exposed to different gravity conditions measured by FACS-analysis.** Non-activated and Concanavalin A (ConA)/CD28-activated CD4+ T lymphocytes were exposed to altered gravity. **A** and **B.** Samples were stained against phosphorylated LAT Y171 (LAT pY171) and analyzed by FACS; data are expressed as relative fluorescence intensity (RFI). **A:** Non-activated (−) T lymphocytes exposed to microgravity (μg) and to an on-board reference centrifuge (1g) showed a significant increase of LAT pY171-staining compared to the cells of the hardware ground control (H/W). LAT pY171-staining was not significantly different between 1g and μg samples. **B:** In ConA/CD28 activated (+) T lymphocytes LAT pY171-staining was not significantly different between 1g and μg samples, although both sample types showed a higher staining than baseline (BL) samples. LAT pY171-staining staining did not differ between BL and H/W samples. Medians and interquartile ranges are shown (* p < 0.1, ** p < 0.05, ns = not significantly different, two-tailed Mann–Whitney-U-Test). **C** and **D.** Samples were stained against phosphorylated LAT Y226 (LAT pY226) and analyzed by FACS; data are expressed as relative fluorescence intensity (RFI). **C:** Non-activated (−) T lymphocytes exposed to microgravity (μg) and to an on-board reference centrifuge (1g) showed a significant increase of LAT pY226-staining compared to the cells of the hardware ground control (H/W). LAT pY226-staining was not significantly different between 1g and μg samples. **D:** In ConA/CD28 activated (+) T lymphocytes LAT pY226-staining was neither significantly different between 1g and μg samples, nor between baseline (BL) samples and 1g or μg samples. LAT pY226-staining increased from culture control samples (CC) to H/W samples and from H/W samples to BL samples. Medians and interquartile ranges are shown. (* p < 0.1, ** p < 0.05, ns = not significantly different, two-tailed Mann–Whitney-U-Test).

Phosphorylation of LAT (linker of activated T cells) is an early event in the cascade of T cell activation. We thus investigated the phosphorylation of LAT at the sites that are phosphorylated during activation: tyrosine-171 and tyrosine-226. In non-activated T cells, staining of pY171-LAT in 1g samples (3.02, 2.97-3.15, n=3) and μg samples (2.89, 2.85-3.03, n=4) was significantly higher than in H/W controls (2.53, 2.43-2.56, n=4, p=0.057 and p=0.029, respectively). Staining of 1g samples and μg samples were not significantly different (p=0.229) (Figure 6A). Staining of pY226-LAT in non-activated T cells revealed that phosphorylation in 1g samples (1.89, 1.88-1.94, n=3) and μg samples (1.83, 1.78-1.92, n=4) was significantly higher than that of H/W controls (1.25, 1.19-1.32, n=4, p=0.057 and 0.029, respectively) (Figure 6C). PY171-LAT was significantly higher in culture controls (5.62, 5.13-5.84, n=4) than in hardware ground controls (4.35, 4.05-4.41, n=4, p=0.029) (Figure 6B). Staining of baseline samples (4.33, 4.11-4.53, n=4) was not different from hardware ground controls (p=0.657).

In the analysis of T cell activation, an enhanced staining intensity (compared to baseline) was observed in both 1g samples (5.11, 4.92-5.24, n=3, p=0.057) and μg samples (5.29, 4.92-5.24, n=3, p=0.057). Direct comparison of 1g samples and μg samples displayed a tendency of a lower signal in 1g samples, although this was tested as being insignificant, p=0.200 (Figure 6B). Staining of pY226-LAT (Figure 6D) was increased in H/W controls (1.50, 1.45-1.62, n=4) compared to CC (1.32, 1.25-1.39, n=4, p=0.029). A further increase was measured in the BL samples compared to H/W controls (1.82, 1.65-1.88, n=4, p= 0.057).

During T cell activation, μg samples (2.26, 1.70-2.33, n=3) and 1g samples 1.69, 1.65-1.81, n=3) neither differed from BL samples (p=0,400 and p=0.571, respectively), nor did they differ from each other (p=0.2) (Figure 6D).

### Phosphorylation of p44/42 MAPK

**Figure 7:**
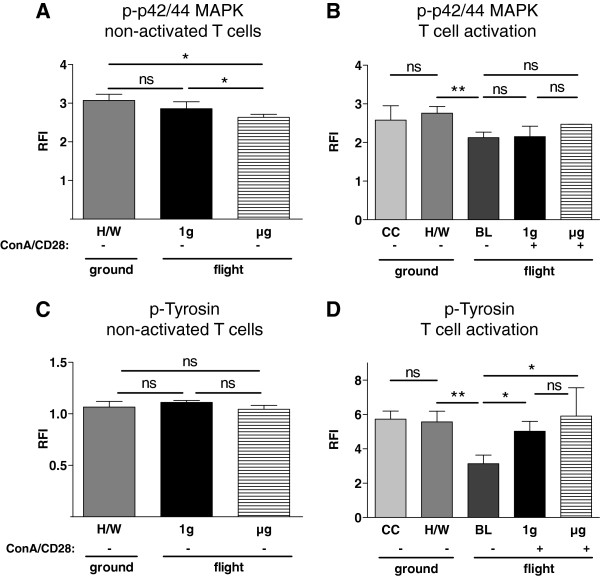
**P-p42/44 MAPK and tyrosine phosphorylation in T lymphocytes exposed to different gravity conditions measured by FACS-analysis.** Non-activated and Concanavalin A (ConA)/CD28-activated CD4+ T lymphocytes were exposed to altered gravity. **A** and **B.** Samples were stained against P-p42/44 MAPK and analyzed by FACS; data are expressed as relative fluorescence intensity (RFI). **A:** Analysis of non-activated (−) T cells exposed to microgravity (μg) showed a significant reduction in RFI in μg samples compared to 1g on board reference samples (1g). **B:** In the analysis of ConA/CD28 activated (+) T lymphocytes a significant reduction of baseline (BL) samples was observed compared to hardware (H/W) samples. The RFIs of 1g samples and μg samples were not significantly different from BL samples or from each other. The medians and individual interquartile ranges are shown. (* p < 0.1, ** p < 0.05, ns = not significantly different, two-tailed Mann–Whitney-U-Test). C and D. Samples were stained against phospho-tyrosine (p-tyrosine) and analyzed by FACS; data are expressed as relative fluorescence intensity (RFI). **C:** Analysis of non-activated (−) T cells revealed no difference between microgravity (μg)-samples, on-board reference centrifuge (1g)-samples and hardware ground controls (H/W) in p-tyrosine stainings. **D:** In the analysis of ConA/CD28 activated (+) T lymphocytes p-tyrosine-staining was not significantly different between 1g and μg samples, but both sample types showed a higher staining than baseline (BL) samples. BL samples were significantly less stained than H/W samples. The medians and individual interquartile ranges are shown. (* p < 0.1, ** p < 0.05, ns = not significantly different, two-tailed Mann–Whitney-U-Test).

The p44/42 mitogen activated protein kinase (p44/42 MAPK) is a cytosolic element of the T cell activation cascade downstream of the lipid-raft-associated signalosome complex of T cell activation. During T cell activation, phosphorylation of p44/42 MAPK leads to the activation of transcription factors such as AP1, which initiates activation-specific gene expression. By directly comparing the 1g samples (2.86, 2.78-3.04, n = 3) with the μg samples (2.64, 2.61-2.71, n=4) a significant reduction of phosphorylated p44/42 MAPK could be detected in non-activated T cells (p = 0.0571) (Figure 7A). Additionally, a significantly lower RFI was observed for the μg samples compared to H/W samples (3.07, 2.88-3.23, n = 4, p = 0.0286). No significant change was found in the 1g samples compared with the H/W samples (p = 0.400) (Figure 7A). No difference between phosphorylated p44/42 MAPK in CC samples and H/W samples was observed (Figure 7B).

During T cell activation, a significant decrease was observed in BL samples (2.13, 2.04-2.24, n=4), compared to H/W samples (2.760, 2.64-2.90, n=4, p=0.086) (Figure 7B). 1g samples (2.15, 1.83-2.42, n=3) and μg samples (2.47, 2.15-2.47, n=3) were not different, neither among each other (p=0.3000) nor compared to the BL samples (Figure 7B).

### Overall tyrosine phosphorylation

T cell activation involves a cascade of phosphorylation, mostly on tyrosine-residues. Therefore, the overall tyrosine-phosphorylation was compared for the different gravity conditions. In non-activated T cells, no difference between the H/W samples (1.065, 1.025-1.12, n=4), 1g samples (1.11, 1.11-1.13, n=3) or μg samples (1.05, 1.02-1.08, n=4) (Figure 7C) was observed, although μg samples had a significantly reduced level of tyrosine-phosphorylation compared to 1g samples (p= 0.0571). P-tyrosine staining was not significantly different between culture controls (5.73, 5.60-6.11, n=4) and hardware ground controls (5.57, 5.22-6.11, n=4, p=0.657) (Figure 7D). Baseline samples (3.14, 2.49-3.55, n=4) demonstrated significantly lower tyrosine-phosphorylation compared to hardware ground controls (p=0.029).

Comparison between baseline samples and μg samples (5.91, 5.26-7.56, n=3, p=0.057) and 1g samples (5.02, 4.09-5.60, n=3, p=0.057) during T cell activation revealed a significant upward regulation. Direct comparison of 1g samples and μg samples displayed a tendency toward a lower signal in 1g samples, although this was tested as not significant (p=0.2).

### Acetylation of histone H3

**Figure 8:**
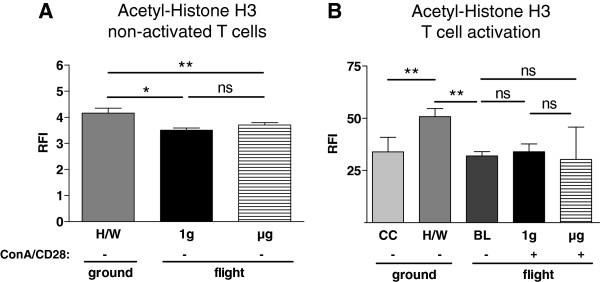
**Acetyl-Histone H3 in T lymphocytes exposed to different gravity conditions measured by FACS-analysis.** Non-activated and Concanavalin A (ConA)/CD28-activated CD4+ T lymphocytes were exposed to altered gravity. Samples were stained against acetyl-histone H3 and analyzed by FACS; data are expressed as relative fluorescence intensity (RFI). **A:** Analysis of non-activated (−) T cells revealed that microgravity (μg)-samples and on-board reference centrifuge (1g)-samples were stained significantly less for acetyl-histone H3 than hardware ground control (H/W)-samples. μg-samples and on-board reference centrifuge (1g)-samples did not differ in their staining intensities. **B:** In ConA/CD28 activated (+) T lymphocytes acetyl-histone H3-staining was neither significantly different between 1g and μg samples, nor between baseline (BL) samples and 1g or μg samples. BL samples were stained significantly less than H/W samples, and H/W samples were stained significantly stronger than culture control (CC) samples. The medians and individual interquartile ranges are shown. (* p < 0.1, ** p < 0.05, ns = not significantly different, two-tailed Mann–Whitney-U-Test).

Since recent findings suggest a possible role of histone acetylation in the transduction of gravitational effects [13], we quantified acetyl-histone H3 in response to altered gravity. In non-activated T cells, both 1g samples (3.50, 3.32-3.59, n=3) and μg samples (3.71, 3.55-3.80, n=4) contained less acetyl-histone H3 compared to H/W controls (4.17, 3.96-4.35, n=4), p=0.057 and p= 0.029 respectively. Staining of μg samples and of 1g samples did not differ significantly in intensity, p=0.075 (Figure 8A).

Acetyl-histon H3 was significantly enhanced in H/W controls (50.77, 43.50-54.12, n=4) compared to culture controls (33.91, 31.15-39.27, n=4), p= 0.029 (Figure 8B). In baseline samples (31.96, 25.25-54.12, n=4) a significantly reduced staining intensity was measured compared to H/W controls, p=0.029. During T cell activation, neither 1g samples (33.97, 32.22-37.74, n=3) nor μg samples (30.28, 26.8-45.78, n=3) had a significantly different staining intensity compared to baseline samples (p=0.400, and p=0.857 respectively) (Figure 8B).

### Expression of vimentin

**Figure 9:**
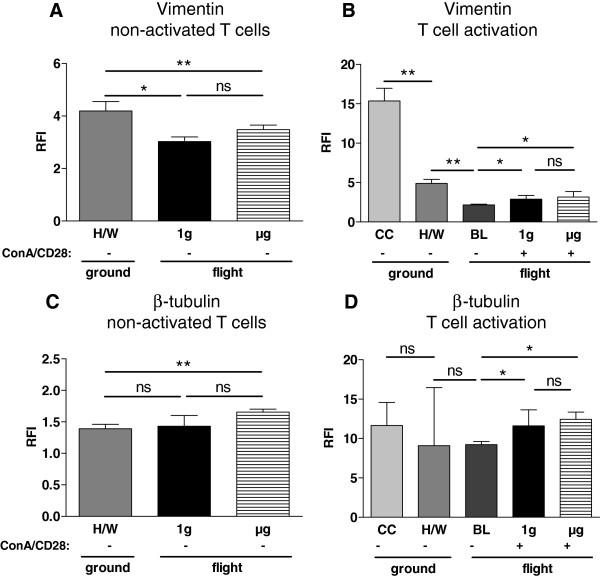
**Vimentin and β-tubulin expression in T lymphocytes exposed to different gravity conditions measured by FACS-analysis.** Non-activated and Concanavalin A (ConA)/CD28-activated CD4+ T lymphocytes were exposed to altered gravity. **A** and **B.** Samples were stained against vimentin and β-tubulin and analyzed by FACS; data are expressed as relative fluorescence intensity (RFI). **A:** Analysis of non-activated (−) T cells revealed that microgravity (μg)-samples and on-board reference centrifuge (1g)-samples were stained significantly less for vimentin than hardware ground control (H/W)-samples. μg-samples and 1g-samples did not differ significantly in their staining intensities. **B:** In ConA/CD28 activated (+) T lymphocytes vimentin-staining was not significantly different between 1g and μg samples, but both samples types are stained significantly stronger than baseline (BL)-samples. BL samples were stained significantly less than H/W samples, and H/W samples were stained significantly less than culture control (CC) samples. The medians and individual interquartile ranges are shown. (* p < 0.1, ** p < 0.05, ns = not significantly different, two-tailed Mann–Whitney-U-Test). **C** and **D.** Samples were stained against β-tubulin and analyzed by FACS; data are expressed as relative fluorescence intensity (RFI) C: Non-activated (−) T lymphocytes exposed to microgravity (μg) showed a significant increase of β-tubulin in comparison to the cells of the flight hardware on ground (H/W). **D:** In the analysis of T cell activation ConA/CD28 activated (+) T lymphocytes both the 1g on-board reference centrifuge (1g) and the microgravity (μg) samples had a significantly higher expression of β-tubulin compared with the cells of the baseline (BL) samples. BL and H/W samples had a lower β-tubulin signal than the culture controls.

Vimentin staining was not different between μg samples (3.49, 3.22-3.66, n=4) and 1g samples (3.03, 2.86-3.20, n=3), p=0.114. In both conditions, staining was significantly decreased compared to H/W controls (4.20, 3.81-4.56, n=4), p= 0.029 compared to μg, and p= 0.057 compared to 1g samples (Figure 9A). Vimentin expression after transfer into the hardware (4.89, 4.44-5.29, n=4) was significantly lower than in culture controls (15.36, 14.59-16.71, n=4, p=0.029). A further significant reduction was measured in comparison between H/W ground controls and baseline samples after the launch phase (2.15, 1.84-2.24, n=4, p=0.0029).

In activated T cells, μg samples (3.16, 2.61-3.86, n=3) and 1g samples (2.89, 2.5-3.36, n=3) exhibited enhanced vimentin staining compared to baseline samples (p= 0.057 and p=0.057 respectively). The direct comparison between μg samples and 1g samples was not significant (p=0.7), although a tendency towards a higher level of staining in μg was visible (Figure 9B).

### Expression of β-tubulin

Analysis of non-activated T cells revealed a significantly higher β-tubulin content in the μg samples (1.66, 1.55-1.70, n = 4) compared to H/W samples (1.39, 1.35-1.46, n = 4) (p = 0.0286), whereas 1g samples (1.43, 1.43-1.60, n = 3) and H/W samples (p = 0.2000) were not different (Figure 9C). A decreased β-tubulin staining in BL samples (9.23, 7.40-9.56, n = 4) compared to H/W samples (9.09, 7.21-14.67, n=4) was observed.

During T cell activation, an increase of β-tubulin in the 1g samples (11.60, 11.22-13.64, n = 3) and the μg samples (12.44, 10.42-13.35, n = 3) was abundant compared to baseline samples (p=0.057 and p=0.075 respectively). In a direct comparison, a significant difference between the 1g samples and the μg samples could not be observed (p = 0.9000) (Figure 9D).

### Distinct alteration in key signal transduction modules due to changes in culture conditions

Table 1 provides a summary of the effects of the hardware, the effects of the launch / hypergravity phase and the microgravity phase. Cultivation of the human primary T lymphocytes in the experiment hardware instead of inside regular cell culture flasks resulted in a downregulation of CD3, ZAP-70, de-phosphorylation of LAT at tyrosine-171 and phosphorylation at tyrosine-226. Acetyl histone H3 increased and vimentin protein was reduced. Thus, important signal molecules were distinctly altered only upon change of the cultivation hardware, despite comparable culture conditions (37°C).

**Table 1:** Summary of the results obtained from the MASER-12 suborbital space flight mission

	**Target molecule**									
	**CD3**	**IL-2R**	**ZAP-70**	**LAT (pY171)**	**LAT (pY226)**	**P-p44/42 MAPK**	**p- tyrosine**	**Acetyl-histone H3**	**Vimentin**	**β-tubulin**
**Effect of cultivation in experiment hardware H/W compared to CC**	↓**	**-**	↓**	↓**	↑**	**-**	**-**	↑**	↓**	**-**
**Effect of launch phase / hypergravity BL compared to H/W**	↓**	↓**	↓*	**-**	↑*	↓**	↓**	↓**	↓**	-
**Effect of microgravity non-activated T cells (μg and 1g compared to H/W)**	↓**	↓**	-	-	-	↓*	-	-	-	-
**Effect of microgravity non-activated T cells (direct comparison μg vs. 1g)**	-	-	-	-	-	↓*	-	-	-	-
**Effect of microgravity ConA/CD28-activated T cells (μg and 1g compared to BL)**	↓*	↓*	-	-	-	-	-	-	-	↑**
**Effect of microgravity ConA/CD28-activated T cells (direct comparison μg vs. 1g)**	-	-	-	-	-	-	-	-	-	-

The effect of cultivation in experiment hardware (H/W compared to CC), the effect of launch phase / hypergravity (BL compared to H/W), the effect of microgravity in non-activated T cells (μg and 1g compared to BL and direct comparison μg vs. 1g) and the effect of microgravity in ConA/CD28-activated T cells (μg and 1g compared to BL and direct comparison μg vs. 1g) are demonstrated. (↑*): increase, p<0.1; (↑**): increase, p<0.05 according to two-tailed Mann–Whitney-U-Test. (↓*): decrease, p<0.1, (↓**): decrease, p<0.05 according to two-tailed Mann–Whitney-U-Test. ( ): no significant changes.

### Hypergravity induced downregulation of IL-2 receptor protein expression, overall tyrosine phosphorylation, p42/44-MAPK phosphorylation and histone H3 acetylation

The launch phase, which provided between 5-7g hypergravity (with a maximum acceleration of 13g) for a period of 43 s, resulted in a downregulation of CD3, the IL-2 receptor, ZAP-70 and phosphorylation of LAT at tyrosine-226. Overall tyrosine-phosphorylation and phosphorylation of p42/44-MAPK was reduced. Acetyl histone H3 increased and vimentin protein was reduced. Thus, downregulation of IL-2 receptor protein expression, overall tyrosine phosphorylation, p42/44-MAPK phosphorylation and histone H3 acetylation cannot be explained by alterations of the cell culture conditions and should therefore be considered as an effect of the launch phase / hypergravity conditions.

### Microgravity reduced CD3 and IL-2 receptor expression and p42/44 MAPK phosphorylation

Compared to the baseline situation at the point of entry into the microgravity phase, CD3 and IL-2 receptor expression at the surface of non-activated T cells were reduced after 6 min microgravity, but not after 6 min 1g (in-flight reference centrifuge). However, the direct comparison between the time points of fixation (6 min after onset of microgravity) revealed no significant differences between the μg and the 1g group. Importantly, p42/44-MAPK-phosphorylation was reduced after 6 min microgravity compared to the baseline situation, but also in direct comparison between the μg and the 1g group. In activated T cells, CD3 and IL-2 receptor expression were reduced also in comparison with the baseline situation, but not significantly different in a direct comparison between the μg and the 1g group. Beta-tubulin increased significantly after onset of microgravity until the end of the microgravity phase, but not during the in-flight 1g condition.

## Discussion

In this study we investigated the influence of altered gravity on key proteins and early signal transduction events of T cell activation. The experiment was performed during the MASER-12 suborbital space mission on board of a ballistic rocket flight with an apogee of 260 km and 390 s microgravity, and with a quality of less than 10 ^-4^ g in all three axis. The experiment hardware “Biology in Microgravity-2” (BIM-2) was specifically developed to match experimental requirements of biomedical research and was designed to maintain tight temperature control during, before and after the flight. Samples were integrated in a late access unit (LAU) that was integrated into the rocket payload only 4 hours before launch and retrieved within 2 hours after landing. The intervals during which the samples were not available before and after flight were extremely short compared to orbital flight missions.

To distinguish the effects of microgravity from the effects due to cell cultivation inside the BIM-2 hardware handling or as the consequence of the launch acceleration, cell culture controls, H/W controls, 1g in-flight centrifuge controls and baseline controls were performed. The 1g in-flight centrifuge control is the standard for discovering microgravity-induced effects, as the samples experienced exactly the same scenario as the μg-samples except for the different gravity condition. The baseline control directly before the onset of microgravity represents the status after launch acceleration and a hypergravity phase of up to 13g. Possible effects of the launch acceleration applied to all on-board samples and must therefore be quantified by the baseline control group and considered for interpretation of the 1g and the μg in-flight group. Although this experiment was conducted using the above-mentioned highest possible standards for experiments on board of a sounding rocket, one must be aware of the fact that all microgravity research platforms are associated with limitations in the sample number, sample volume and sample accessibility. It is unavoidable that cells are exposed to conditions that deviate from the cell culture conditions in a ground laboratory. Therefore, results from the sounding rocket experiments should be interpreted very carefully in the light of appropriate control experiments. Although receptor-specific stimulation models other than ConA/CD28 are available [13], this model was chosen as an established system for T cell stimulation in microgravity experiments, because it has been used several times before [7,25-27] and thus provided the possibility of integrating the knowledge from previous studies in the interpretation of our results. Our study clearly indicated that even a change of the culture material (from standard cell culture flasks to a biocompatible experiment hardware) was sufficient to induce distinct changes in the signal transduction network such as downregulation of CD3 and ZAP-70, de-phosphorylation of LAT at tyrosine-171 and phosphorylation at tyrosine-226 and increase of acetyl histone H3.

Additionally to the hypergravity induced by launch acceleration, possible vibration effects should be considered. Vibration can trigger inflammatory cascades, as reflected by the increase in IL-8 release mediated by MAPK pathways [28]. Other experiments could not demonstrate vibration effects in T cells: Hsp70 and hsp27 mRNA were not up-regulated, although an up-regulation was seen in spaceflight [29], and low frequency vibrations induced no cytogenetic effects in human blood lymphocytes [30]. The cell compartment was enclosed completely by a thick silicon membrane, which was encased in a plastic housing, located inside the hardware cassette. Thus, the cell culture system was very well dampened against vibrations. During the initial launch phase, hypergravity forces due to the rocket acceleration were accompanied by launch vibration and a spin rate of 3–4 Hz. After second stage burn out and de-spin maneuver, a high-quality microgravity environment with less then10E-4 g in all three axis and with no vibration was achieved. Thus, biological phenomena detected in the “baseline” samples directly before entry into the microgravity phase, could represent mainly effects of hypergravity and vibration, whereas during the microgravity phase, vibration effects can be excluded to a large extent.

We found that p42/44-MAPK-phosphorylation was reduced in non-activated T cells after 6 min microgravity in direct comparison between the μg and the 1g group and compared to the baseline situation, but not in activated T cells. Decreased p44/42 MAPK phosphorylation also occurred during the launch acceleration in a comparison between the H/W samples and BL samples. Because no difference was detected between the cell culture control group (CC) and the H/W group, the alterations are not the consequence of cell culture conditions, but rather the result of the altered gravity. Surprisingly, p44/42 MAPK phosphorylation was not observed after ConA/CD28-activation in the 1g in-flight control group compared to the BL samples. This could be the consequence of the preceding hypergravity phase, which obviously activated p44/42 MAPK de-phosphorylating pathways and might have prevented p44/42 MAPK phosphorylation during the subsequent ConA/CD28-activation. In contrast, 5 min simulated microgravity by 2D clinorotation led to an increase of phosphorylated p44/42 MAPK in non-activated Jurkat T cells as well as in CD3/CD28-activated Jurkat T cells [15]. However, the experimental conditions are not comparable because of the different T cell types and particularly because of the preceding strong acceleration phase during the MASER-12 launch. The distinct increase in overall tyrosine phosphorylation after addition of ConA/CD28 at the onset of microgravity in both μg and 1g samples demonstrated the successful activation of T cells (Figure 7D). Neither in non-activated nor in activated T cells was tyrosine phosphorylation different between μg and 1g. Previous experiments in simulated microgravity provided by the 2D clinostat [15] or the rotating wall vessel (RWV) [31] also revealed no changes in overall protein phosphorylation in T cells. Therefore, we consider de-phosphorylation of p42/44 MAPK in microgravity as a specific effect and not as part of a general effect on tyrosine phosphorylation.

Physiologically the TCR/CD3 complex in naive T cells is constantly internalized and recycled, resulting in a constant level of CD3 surface expression [32]. The reduced surface expression of CD3 after the hypergravity phase could therefore be the consequence of an enhanced internalisation of CD3 or of a decreased recycling of the TCR-CD3 receptor. After a reduction during the hypergravity phase (comparison between H/W and BL), CD3 surface expression reconstituted after T cell activation in the 1g in-flight group, but not in the μg group. Since the TCR/CD3 complex decreased in response to antigen-binding [33,34], an increase after ConA/CD28 activation could be explained as counter-regulation after the strong initial launch-phase induced down-regulation (Figure 4B). Thus, such counter-regulatory processes aiming to restore the “physiological” situation could be disturbed in microgravity. In a previous study it was reported that after CD3 stimulation, CD3 internalisation was slower during clinorotation than in 1g samples [35]. Therefore, the dynamics of CD3 receptor internalization and recycling is obviously influenced by altered gravity.

IL-2 receptor surface protein was down-regulated during the launch phase, but not after transfer into the experimental hardware, and can therefore be considered as an effect of the launch / hypergravity conditions. Compared to the baseline situation at the point of entry into the microgravity phase, IL-2 receptor expression at the surface of non-activated T cells was reconstituted significantly during 1g, but not significantly during microgravity conditions, whereas direct comparison between μg and 1g at the time points of fixation revealed no significant differences. However, non-significant levels in the BL vs. μg group could be also the consequence of the limited sample size. Therefore, whereas IL-2 receptor surface expression is obviously sensitive to hypergravity, an effect of microgravity could not be demonstrated clearly. Because IL-2 receptor mRNA expression was down-regulated in simulated microgravity [12,21,22], this transcriptional effect obviously could still not be translated into changes of the protein status during 6 min.

Rapid changes in surface receptors could be the result of cytoskeletal alterations and dynamics. We thus investigated β-tubulin as a component of microtubules, and vimentin as an important intermediate filament protein. Whereas vimentin protein was reduced after the launch phase, no difference was detected between 1g and μg samples for vimentin or for β-tubulin. Rapid changes of the cytoskeleton as reaction to gravitational changes were reported in diverse cell types [36-40]. As demonstrated during a MAXUS suborbital rocket mission, the vimentin network was strongly impaired in Jurkat T cells during microgravity [41]. Interestingly, the most distinct decrease occurred after cultivation in the experimental hardware, which underlines the paramount importance of appropriate hardware controls in microgravity experiments.

Results from gravitational effect on T cells have been reviewed before [42]: In early experiments conducted on several space missions (Spacelab 1 in 1983, Spacelab D-1 in 1985, Spacelab Life Science-1 in 1991, International Microgravity Laboratory-2 in 1994) mitogenic activation of T cell with ConA was determined. In those cases the cell cultures contained purified lymphocytes, namely T and B cells together with monocytes as accessory cells. The mitotic index determined 3 days after activation was constantly depressed by 60-90% in the μg samples compared to the 1 g controls. In more recent experiments [21], purified cultures of T cells were activated with ConA/CD28 as a substitute for the accessory cells, and gene expression was determined 2–4 h after activation. Due to the tragic loss of the Space Shuttle Columbia in 1993, for which these investigations had been planned, the experiments were performed using a random positioning machine (RPM) and impaired induction of early genes regulated primarily by transcription factors NF-κB, CREB, ELK, AP-1 and STAT were discovered, while PKA was suggested as a key player in the loss of T-cell activation in altered gravity [21]. In a study conducted aboard the International Space Station (ISS) in 2006 we tested the hypothesis that transcription of immediate early genes is inhibited in T cells activated with ConA/CD28 in microgravity [43]. Microarray expression analysis after 1.5 h of activation demonstrated that 47 genes were significantly down-regulated differentially in microgravity. Importantly, transactivation of Rel/NF-κB, CREB and SRF gene targets was down-regulated, expression of cREL gene targets was significantly inhibited, and transcription of cREL itself was reduced significantly [43]. These experiments indicated that transcriptional effects of microgravity could be the cause of impaired T cell activation during spaceflight [43]. Importantly, in this study on board of the ISS, where the same cell type and activation procedure as during the MASER-12-flight was used (10 μg/ml ConA / 4 μg/ml anti CD-28), several results supported a key role of MAPK-dependent signaling in T cells activated in microgravity: The distinct inhibition of DUSP2 expression (dual-specificity phosphatase 2), a phosphatase which dephosphorylates MAPK, and the high representation of CREB1-binding sites in the promoter region of inhibited genes, which can be activated by Ras/MAPK-dependent pathways.

Cellular and gene expression adaptation effects to low gravity in cells of the immune and the hematopoietic system were described and discussed recently [44-46] and were also the subject for computer modeling studies [47]. Additionally, there are some indications that cells can be conditioned to a different gravity environment: Whereas human lymphoid cells, cultivated either in a rotating culture vessel or in microgravity in the ISS, did not respond to antigenic or polyclonal challenge, they maintained their antibody and cytokine responses in space if challenged prior to microgravity exposure [48]. Additionally, functional immune dysregulation varied related to mission duration [49]. Thus, investigation of adaptation and pre-conditioning mechanisms are necessary and important. However, due to space limitations and the technical design of the BIM-2 module, fixation after different time points of the microgravity phase was technically not possible during the MASER-12 experiment. The sample size was already at the lower limit and important controls could not be removed. The MASER-12 experiment will be continued during a parabolic flight campaign, with exactly the same experimental conditions, to answer the question if rapid adaption occurred after minutes of microgravity or if the investigated signal molecules were not affected at all. However, from the results of our previous ground-based studies using 2D clinorotation [15], we can support the hypothesis of adaptation effects: Phosphorylation of ERK-1/2 in Jurkat T cells was enhanced after 1min simulated weightlessness compared to 1g controls, oscillated between 2min and 10min and disappeared also after 15min. It seems that MAPK activation in T cells is a rapid, but transient response to simulated weightlessness. In further previous experiments with T cells incubated in the 2D clinostat, we found that the expression of proteins associated with cell cycle regulation changed significantly in the time course of 15min [13]. Thus, end-point measurements such as during the MASER-12 mission should be interpreted only in the context of experiments in real microgravity with other time points and supporting ground-based experiments using the 2D clinostat. We recommend to address preconditioning and adaptation effects in further studies. Hyper-gravity preconditioning can be investigated *in vitro* by cell culture centrifuges, but also *in vivo* by the short arm human centrifuge (SAHC) [50] or maybe in the future in more sophisticated settings such as the “Large Human Centrifuge” [51].

This study indicates that the key proteins of T cell signal modules were not severely disturbed in microgravity. Instead, we suppose that the strong T cell inhibiting signal occurs downstream from membrane proximal signaling, such as at the transcriptional level as described recently [13,39,40]. Nevertheless, the MASER-12 experiment could identify signal molecules which are sensitive to altered gravity. It indicated that gravity is obviously not only a requirement for transcriptional processes as described previously, but also for specific phosphorylation / dephosphorylation of signal molecules such as p42/44 MAPK and surface receptor dynamics. We therefore suppose that the specific gravity on Earth could be one of the fundamental conditions of signal transduction structures in mammalian cells.

## Abbreviations

BIM-2: Biology In Microgravity-2; CCM: Centre for Concepts of Mechatronics; ConA: Concanavalin A; ESA: European Space Agency; ESRANGE: European Space and Sounding Rocket Range; H/W: Hardware; IL-2: Interleukin-2; IL-2R: Interleukin-2 receptor; LAU: Late Access Unit; LAT: Linker of activation of T cells; MAPK: Mitogen-activated protein kinase; PBMC: Peripheral blood mononuclear cells; RFI: Relative fluorescence intensity; SSC: Swedish Space Corporation; ZAP: Zeta-chain-associated protein kinase70.

## Competing interests

The authors declare that they have no competing interests.

## Authors’ contributions

OU, AC and PP developed the study idea, concept and the overall study design in addition to planning, coordinating and supervising the study. ST and OU wrote and edited the manuscript. SH, CC, CS and AC contributed to the manuscript. CC and CS carried out the pre-flight procedures and ST, SH and IB the post-flight procedures (including sample analysis) of the MASER-12 mission. ST and SH also contributed to the pre-flight procedures, and developed and tested the analysis procedures. CC, SC, AP and AS performed the biocompatibility and hardware tests and developed the pre-flight procedures. KP and CT contributed to the sample analysis and mission preparation. All authors read and approved the final manuscript.
